# Engineered Newcastle disease virus expressing the F and G proteins of AMPV-C confers protection against challenges in turkeys

**DOI:** 10.1038/s41598-017-04267-7

**Published:** 2017-06-22

**Authors:** Haixia Hu, Jason P. Roth, Laszlo Zsak, Qingzhong Yu

**Affiliations:** 1 College of Animal Science and Technology, Southwest University, Chongqing, 400715 China; 2 Southeast Poultry Research Laboratory, US National Poultry Research Center, Agricultural Research Services, United States Department of Agriculture, 934 College Station Road, Athens, GA 30605 USA; 3 Merial Ltd., 1730 Olympic Dr., Athens, GA 30601 USA

## Abstract

Avian metapneumovirus (AMPV) infects the respiratory and reproductive tracts of domestic poultry, resulting in substantial economic losses for producers. Live attenuated vaccines appear to be the most effective in countries where the disease is prevalent. However, reversion to virulence has been demonstrated in several studies. Therefore, the development of a stable and safe next generation vaccine against the AMPV disease is needed. In the present study, we generated a recombinant Newcastle disease virus (NDV) vectoring the fusion (F) protein and glycoprotein (G) genes of AMPV subtype-C (AMPV-C) as a bivalent vaccine candidate using reverse genetics technology. The recombinant virus, rLS/AMPV-C F&G, was slightly attenuated *in vivo*, yet maintained similar characteristics *in vitro* when compared to the parental LaSota virus. Vaccination of turkeys with rLS/AMPV-C F&G induced both AMPV-C and NDV-specific antibody responses, and provided significant protection against pathogenic AMPV-C challenge and complete protection against velogenic NDV challenge. These results suggest that the rLS/AMPV-C F&G recombinant virus is a safe and effective bivalent vaccine candidate and that the expression of both F and G proteins of AMPV-C induces a protective response against the AMPV-C disease.

## Introduction

Avian metapneumovirus (AMPV) causes turkey rhinotracheitis (TRT), an acute upper respiratory tract infection in turkeys, and is associated with swollen head syndrome (SHS) in chickens, resulting in substantial economic losses to the poultry industry^1, 2^. Turkey rhinotracheitis was first reported in the late 1970s in South Africa^3^ and since then, the virus has spread to all major poultry-producing areas in the world, except for Australia^2^. Isolates of AMPV have been classified into four subtypes, A, B, C, and D, based on the level of genetic variations and antigenic differences^4–8^. Subtypes A and B are present in most countries in the world, excluding the USA. However, AMPV-C is present in the USA, some Asian countries, and, to a limited degree, France. Finally, AMPV-D has only been isolated in France to-date.

AMPV is a non-segmented, single-stranded negative sense RNA virus, and belongs to the genus *Metapneumovirus* within the family *Pneumoviridae* ^9^. AMPV genome consists of eight genes flanked by a 3′ Leader and 5′ Trailer in the order 3′-N (nucleocapsid)-P (phosphoprotein)-M (matrix)-F (fusion)-M2 (second matrix)-SH (small hydrophobic)-G (glycoprotein)-L (large polymerase)-5′ ^10, 11^. The fusion (F) protein and glycoprotein (G) are two major membrane-associated structure proteins of AMPV, and are believed to play important roles in virus pathogenicity and immunogenicity^6, 12–16^.

Live attenuated vaccines have been developed through serial passaging AMPV field isolates in cell culture^17–19^. Although these live vaccines appear to be effective where the disease is prevalent, the stability and safety of some of these live vaccines are of concern^20–22^. Outbreaks of TRT at vaccinated turkey farms have been reported and were found to be due to the reversion of the vaccine virus after shedding to naïve birds^20, 22, 23^. In addition, it was found that viral subpopulations in AMPV vaccines may not be the only cause of reversion^24^. Therefore, the development of a safe, stable, and readily administered next generation vaccine against AMPV disease is needed.

Previously, we generated Newcastle disease virus (NDV) LaSota vaccine strain-based recombinant viruses expressing the glycoprotein (G) of AMPV-A, B, and C viruses as bivalent vaccines^13, 25^. Although these vaccine candidates were stable and safe in embryonated chicken eggs and vaccinated turkeys, they conferred only partial protection against homologous AMPV challenge in turkeys. It suggests that the G protein alone may be a weak antigen and other viral immunogenic components may be needed in combination to improve efficacy against AMPV diseases. Presence of the other major AMPV surface glycoprotein, fusion (F) protein, has demonstrated protective immunity in turkeys when expressed by either DNA vaccine or recombinant fowlpox virus^14, 26^. Therefore, in the present study, we re-engineered the recombinant LaSota/AMPV-C G virus^13, 25^ to express the F protein of AMPV-C, in combination, in an effort to improve the vaccine efficacy against AMPV-C disease.

## Results

### Generation of the rLS/AMPV-C F&G virus

**Figure 1:**
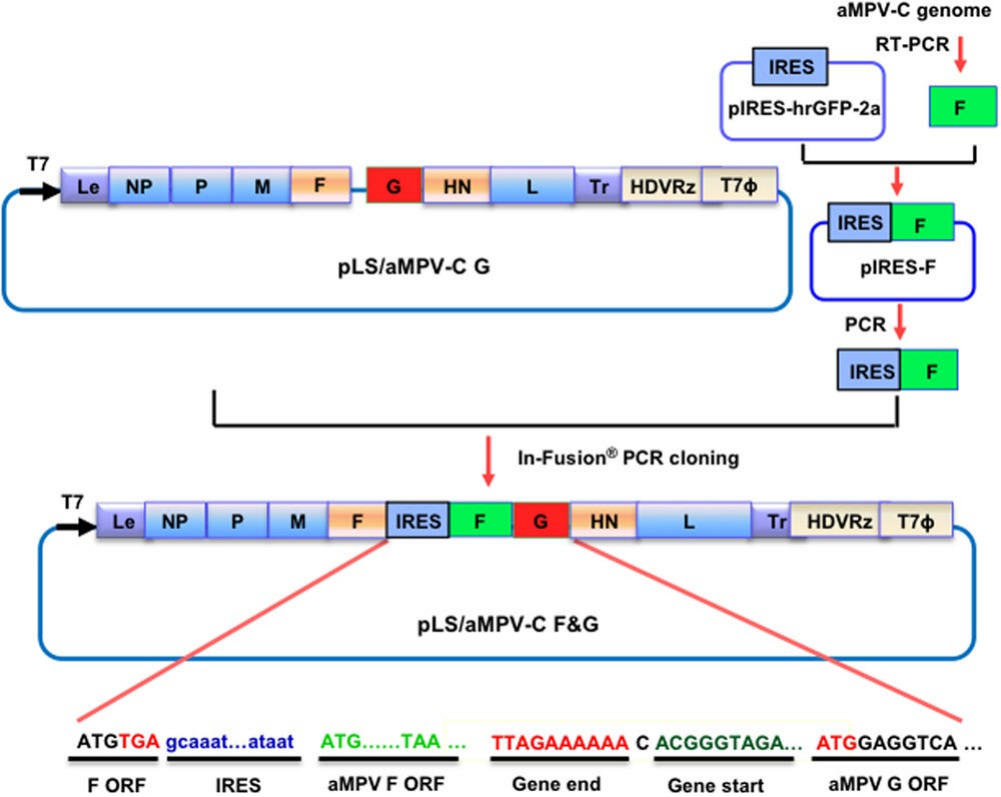
Schematic representation of pLS/AMPV-C F&G construction. The open reading frame of the AMPV-C F gene, amplified from viral genomic RNA, was cloned into the pIRES-hrGFP-2a vector (pIRES, Clontech, Mountain View, CA) downstream of the IRES sequence. Subsequently, the IRES and the AMPV-C F ORF sequences were amplified and cloned into the pLS/AMPV-C G vector downstream of the NDV F ORF using an In-Fusion PCR Cloning Kit (Clontech). The NDV Gene Start and Gene End signal sequences and the AMPV-C F and G ORF sequences are underlined. The direction of the T7 promoter is indicated by a bold black arrow. HDVRz and T7Φ represent the site of the Hepatitis delta virus ribozyme and the T7 terminator sequences, respectively.

A full-length cDNA clone encoding the complete antisense genome of the NDV LaSota vaccine strain and the F and G genes of AMPV-C inserted between the F and HN genes of NDV was constructed using molecular biology techniques (Fig. 1). The resulting cDNA clone, pLS/AMPV-C F&G, was co-transfected into HEp-2 cells, along with supporting plasmids, to rescue an infectious virus. After amplification in SPF chicken embryonated eggs, the LaSota strain-based recombinant virus vectoring the F and G genes of AMPV-C, designated as rLS/AMPV-C F&G, was purified and propagated. The fidelity of the rescued virus was confirmed by sequence analysis of the amplified viral genome (data not shown).

### Biological characterization of the rLS/AMPV-C F&G virus

**Figure 2:**
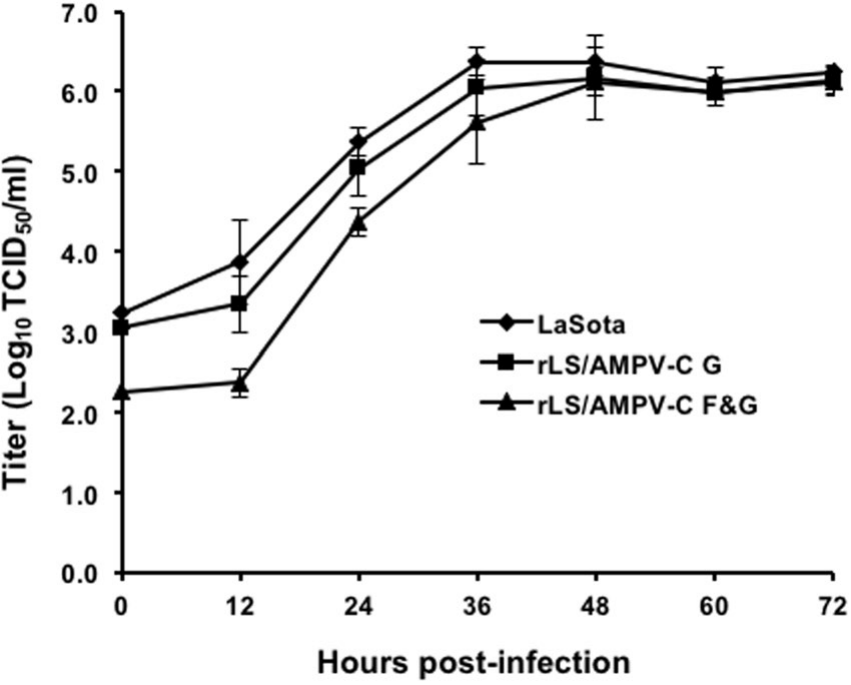
Growth dynamics of the recombinant viruses. DF-1 cells were infected with either rLS/AMPV-C F&G, rLS/AMPV-C G or LaSota strain at an MOI of 0.01. Every 12 h post-infection, virus was harvested by freeze-thaw cycles. Viral titers at each time point were determined by TCID_50_ titration in DF-1 cells, in triplicates. The mean titer at each time point is expressed in log_10_ TCID_50_/ml.

To determine if the addition of the AMPV-C F gene affected viral pathogenicity and growth dynamics, the characteristics of the rLS/AMPV-C F&G virus were examined *in vitro* and *in vivo* using the MDT and ICPI tests and several titration assays. As shown in Table 1, the recombinant viruses appear to be slightly attenuated in day-old chickens with a lower ICPI (0.0) compared to the parental LaSota strain. The titers of the recombinant viruses grown in embryonated eggs or DF-1 cells, measured by EID_50_, TCID_50_, and HA, were comparable to the titers of the parental LaSota strain (Table 1). Replication of the rLS/AMPV-C F&G virus was slightly delayed in the early stages (first 36 hours) of infection, but after time, was able to replicate to similar titers compared to the parental LaSota virus (Fig. 2).

**Table 1:** Biological assessments of the NDV recombinant virus.

Virus	MDT^a^	ICPI^b^	HA^c^	EID_50_^d^	TCID_50_^e^
LaSota	110hs	0.15	1024	6.8 × 10^8^	3.5 × 10^7^
rLS/AMPV-C G	>150hs	0	512	3.2 × 10^8^	1.8 × 10^7^
rLS/AMPV-C F&G	>150hs	0	512	1.5 × 10^8^	1.8 × 10^7^

^a^MDT: Mean death time assay in embryonated chicken eggs.

^b^ICPI: Intracerebral pathogenicity index assay in day-old chickens.

^c^HA: Hemagglutination assay.

^d^EID_50_: 50% egg infective dose assay in embryonated chicken eggs.

^e^TCID_50_: 50% tissue infectious dose assay in DF-1 cells.

### Expression of the F protein by rLS/AMPV-C F&G

**Figure 3:**
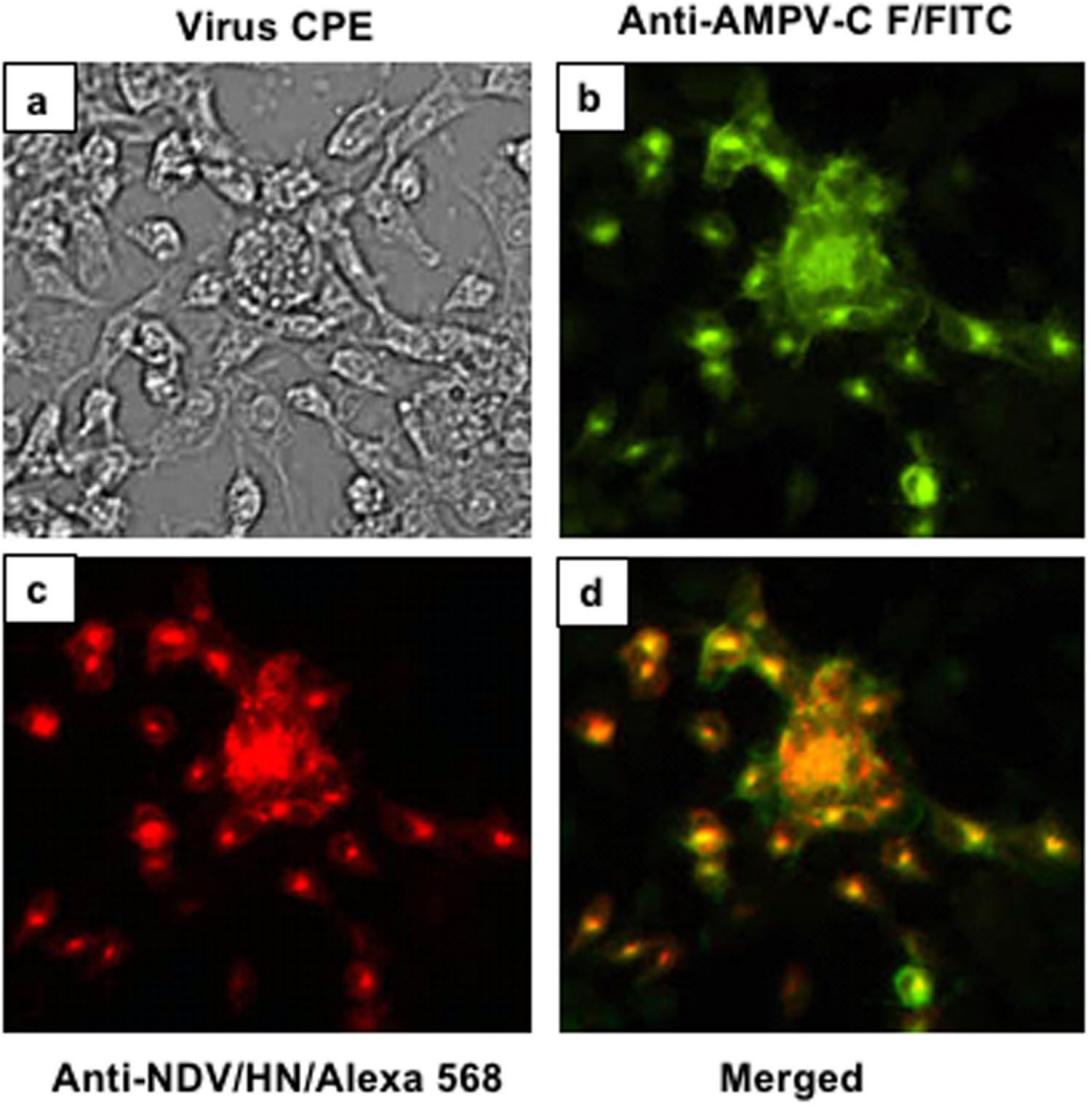
Detection of AMPV-C F protein expression by IFA. DF-1 cells were infected with rLS/AMPV-C F&G at an MOI of 0.01. At 24 h post-infection, the infected cells were fixed and stained with a mixture of chicken anti-AMPV-C F serum and mouse anti-NDV HN Mab followed by staining with a mixture of anti-chicken FITC and anti-mouse Alexa Fluor 568 conjugated antibodies. Stained cells were examined and digitally photographed under 100× magnification. Detection of AMPV-C (**b**) and NDV (**c**) in rLS/AMPV-C F&G-infected cells were merged (**d**). Viral induced CPE from rLS/AMPV-C F&G virus was photographed (**a**) under bright-field.

Expression the AMPV-C G protein from the rLS/AMPV-C G, the parental virus of rLS/AMPV-C F&G, was detected previously^13^. Here we detected expression of the AMPV-C F protein from rLS/AMPV-C F&G infected DF-1 cells by IFA using chicken anti-AMPV-C F polyclonal antibody and the FITC-labeled goat anti-chicken IgG (Fig. 3b). To localize the AMPV-C F expression in relation to the NDV backbone, NDV HN protein expression was also detected by IFA using a mouse anti-NDV HN monoclonal antibody (Mab) and Alexa Fluor 568 conjugated goat anti-mouse IgG (Fig. 3c). After merging both fluorescent images, AMPV-C F and NDV HN protein expression co-localized to the same cells (Fig. 3d), which corresponded to typical NDV induced viral cytopathic effects (CPE) observed under bright-field microscopy (Fig. 3a). This confirms that the AMPV-C F protein was expressed during recombinant virus replication.

### Immune response and protection against AMPV-C challenge

**Figure 4:**
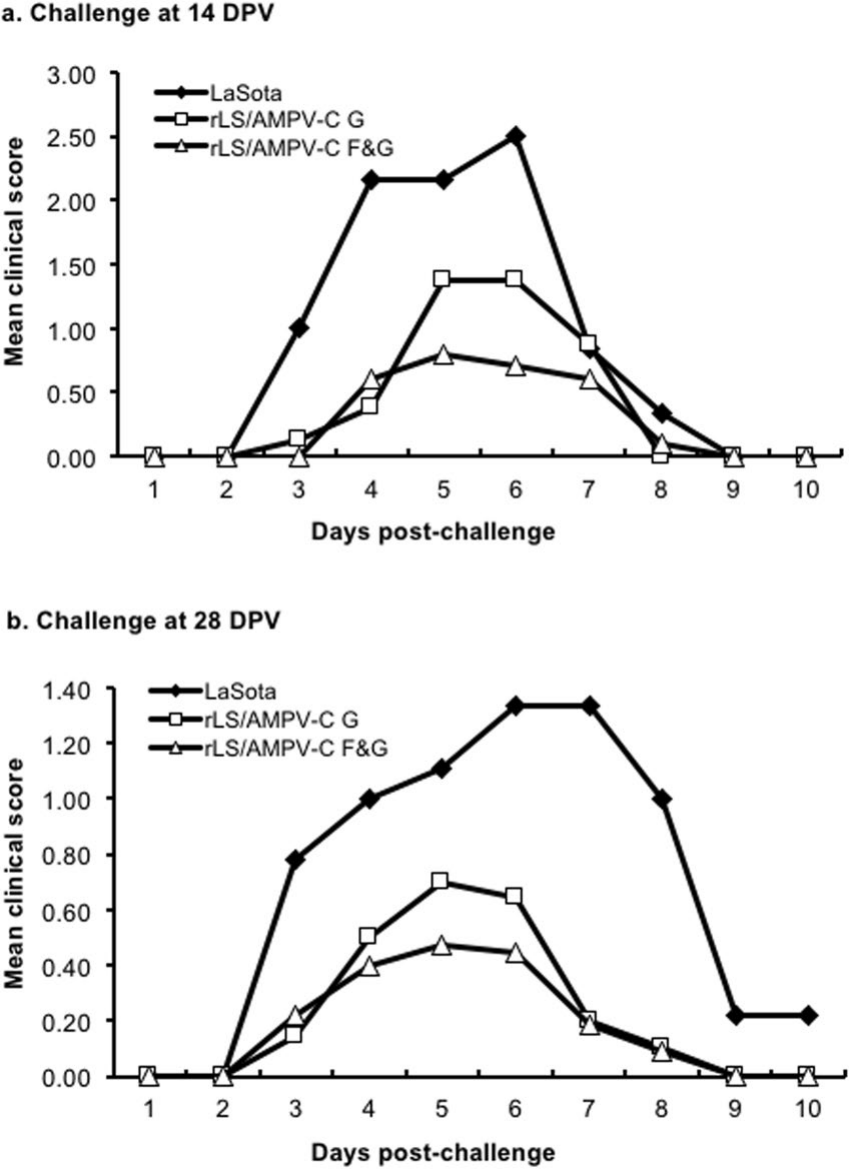
Mean clinical sign scores of turkeys after challenge with AMPV-C. Turkey poults were vaccinated with rLS/AMPV-C G, rLS/AMPV-C F&G, or LaSota, and challenged with the pathogenic AMPV-C at 14 (**a**) or 28 (**b**) DPV. The birds were examined daily for clinical signs and the mean scores from each group were plotted.

To assess the efficacy of each recombinant vaccine, turkeys were vaccinated with each recombinant virus and challenged with AMPV-C. No visible clinical signs were observed in turkeys in any treatment group post vaccination. Sixty percent (60%) of SPF turkeys that were immunized with the rLS/AMPV-C F&G virus produced AMPV-C-specific antibodies at 14 DPV and 100% at 28 DPV as measured by ELISA (Table 2). In contrast, only 40–50% of SPF turkeys vaccinated with the rLS/AMPV-C G virus developed AMPV-C-specific antibodies at 14 and 28 DPV (Table 2). From 3 DPC, 100% of the SPF turkey poults vaccinated with the LaSota control exhibited typical clinical signs of AMPV-C infection when challenged (Fig. 4), showing nasal exudates when squeezed (score 1), nasal discharge (score 2), and/or frothy eyes (score 3). The infected birds showed peak clinical signs between 6–7 DPC that gradually disappeared by 10 DPC. In the rLS/AMPV-C G vaccinated groups, 80% of turkeys showed clinical signs with mean scores of 1.5 (14 DPV challenge, Fig. 4a) and 0.6 (28 DPV challenge, Fig. 4b) for 5–6 DPC, respectively, indicating a partial protection. Whereas 50% of the turkeys in the rLS/AMPV-C F&G vaccinated groups did not show AMPV induced clinical signs when challenged after either 14 or 28 DPV. The remaining birds in the rLS/AMPV-C F&G vaccine groups displayed mild transient clinical signs with lower mean scores of 0.75 for 14 DPV (Fig. 4a) and 0.4 for 28 DPV (Fig. 4b) compared to the rLS/AMPV-C G vaccine groups during peak clinical signs, demonstrating an improvement in protection against AMPV-C infection.

**Table 2:** Serum antibody response against AMPV-C following vaccination and AMPV-C virus shedding in trachea following challenge.

Group	Treatment	Positive Ab sera by ELISA (# of birds)		Viral RNA shedding (# of birds)		
		14 DPV^a^	28 DPV	3 DPC^b^	5 DPC	7 DPC
1	LaSota	0/10^d^	N.D.^c^	10/10^e^	10/10	10/10
3	rLS/AMPV-C G	4/10	N.D.	10/10	10/10	8/10
5	rLS/AMPV-C F&G	7/10	N.D.	10/10	10/10	6/10
2	LaSota	0/10	0/10	10/10	10/10	10/10
4	rLS/AMPV-C G	4/10	5/10	10/10	9/10	7/10
6	rLS/AMPV-C F&G	6/10	10/10	10/10	8/10	2/10

^a^DPV: days post-vaccination.

^b^DPC: days post-challenge.

^c^N.D.: not determined.

^d^Number of birds shedding viral RNA/total number of birds tested.

^e^Number of birds with positive ab/total number of birds tested.

### AMPV-C shedding from vaccinated turkeys after challenge

Viral RNA shedding, or the presence of the challenge virus (AMPV-C) in the tracheal lumen, was detected in 100% of birds in the parental LaSota vaccine control groups at 3, 5, and 7 DPC (Table 2). Challenge virus shedding from turkeys vaccinated with either rLS/AMPV-C G or rLS/AMPV-C F&G was also detected on 3 and 5 DPC. By 7 DPC, the number of vaccinated birds shedding the challenge virus was decreasing, where 75% of turkeys in the rLS/AMPV-C G vaccinated groups and 40% of the birds in the rLS/AMPV-C F&G groups were positive for viral RNA (Table 2). This indicates that the rLS/AMPV-C F&G vaccine may induce better protection against challenge compared to the rLS/AMPV-C G vaccine.

### Immune response and protection against NDV challenge

As shown in Table 3, turkeys immunized with the LaSota vectored and non-vectored vaccines induced a NDV-specific humoral response as measured by HI assay. All vaccinated turkeys were protected against a lethal NDV challenge without showing any signs of disease. In contrast, all of the birds in the unvaccinated control group displayed signs of disease with conjunctivitis and severe depression from 2 to 4 DPC and 100% mortality at 5 DPC (Table 3).

**Table 3:** Serum antibody response against NDV following vaccination and survival of turkeys after challenge with a lethal dose of NDV/CA02.

Treatment	Antibody response		Survivors
	Seropositive birds	HI titer^a^	
PBS	0/10^b^	0	0/10^c^
LaSota	10/10	4.8 ± 1.2	10/10
rLS/AMPV-C G	10/10	3.2 ± 1.7	10/10
rLS/AMPV-C F&G	10/10	3.9 ± 1.4	10/10

^a^Hemagglutination inhibition (HI) titer was expressed in log_2_ of the mean ± standard deviation.

^b^Number of seropositive birds/total number of birds tested.

^c^Number of died birds/total number of birds challenged.

## Discussion

Previously, we generated and evaluated NDV LaSota strain-based recombinant viruses expressing the AMPV G protein as bivalent vaccines, which provided partial protection against homologous AMPV challenge in turkeys^13, 25^. In the present study, we further engineered the recombinant LaSota/AMPV-C G virus to express the other major surface glycoprotein of AMPV-C, the F protein, using an IRES to promote the foreign gene expression within a native NDV transcription unit^27^. This recombinant virus, rLS/AMPV-C F&G, was slightly attenuated in young chickens and delayed the onset of infection in cell culture when compared to the parental LaSota strain. It was stable and did not show any apparent biological changes when measured by MDT and virus titration after 10 passages in SPF chicken embryos. These results demonstrate the safety, stability, and possible application of this recombinant vaccine candidate in young turkeys, which is the most vulnerable population for NDV and AMPV diseases^4, 28, 29^.

The F and G proteins are two major surface glycoproteins of AMPV and believed to play important roles in virus infection, pathogenicity and immunogenicity. Expression of the F or G protein as a vaccine antigen from DNA vaccine, fowlpox or NDV vector conferred only partial protection in homologous challenged turkeys^13, 14, 25, 26^. A combination of the F, G, and/or other antigenic conponents of the virus may be needed to improve the immune response and confer protection. In this study, we showed that the NDV recombinant vectoring both the AMPV-C F and G protein genes, rLS/AMPV-C F&G, induced a stronger immune response in turkeys against AMPV-C challenge than the rLS/AMPV-C G virus expressing the G protein alone. All turkeys vaccinated with the rLS/AMPV-C F&G virus developed AMPV-C specific antibody by 28 DPV, whereas only 50% of the rLS/AMPV-V G virus-vaccinated turkeys seroconverted by this time. Turkeys vaccinated with the F&G virus showed less clinical signs and shed less challenge virus than birds in the control groups at 7 DPC, demonstrating the improved efficacy induced by the presence of both AMPV-C F and G antigens. However, less than 50% the turkeys vaccinated with the rLS/AMPV-C F&G virus still showed mild transient clinical signs between 5–7 DPC. This incomplete protection conferred by the rLS/AMPV F&G virus warrants the need to futher develop a recombinant AMPV-C vaccine candidate and program, perhaps by either relocating the AMPVgenes closer to the 3′ end of the NDV genome for increased expression of the antigens or employing a prime and boost vaccination stragegy.

In summary, in the present study we successfully generated a NDV LaSota strain-based recombinant virus vectoring the F and G protein genes of AMPV-C. Turkeys vaccinated with this recombinant virus were protected against a lethal velogenic NDV challenge and partially protected against pathogenic AMPV-C challenge. Compared with the rLS/AMPV-C G virus, the addition of the F gene in the new recombinant vaccine candidate, rLS/AMPV-C F&G, induced a stronger immune response in turkeys, indicated by reduced clinical signs and viral shedding after challenge. The results suggest that the rLS/AMPV-C F&G may be a promising bivalent vaccine candidate against NDV and AMPV-C disease, yet needs further development before use in large clinical trials on a commercial scale.

## Methods

All methods used in this study were carried out in accordance with the guidelines and protocols approved by the Institutional Biosafety Committee (IBC), Southeast Poultry Research Laboratory, United States Department of Agriculture, Agriculture Research Service (SEPRL, USDA-ARS, Athens, GA, USA). All animal experimental protocols were approved by the Institutional Animal Care and Use Committee (IACUC), SEPRL, USDA-ARS (Athens, GA, USA).

### Cells, viruses and RNA preparation

HEp-2 (CCL-81; ATCC) and DF-1 (CRL-12203; ATCC) cell lines were grown in Dulbecco’s Modified Eagle Medium (DMEM, Life Technologies, Carlsbad, CA) supplemented with 10% fetal bovine serum (FBS, Life Technologies) and antibiotics (100 U/ml Penicillin, 100 µg/ml Streptomycin, 0.25 µg/ml Amphotericin B, Thermo Scientific). The DF-1 cells were maintained at 37 °C and 5% CO_2_ in DMEM supplemented with 10% allantoic fluid (AF) from 10-day-old specific-pathogen-free (SPF) chicken embryos for all subsequent infections unless otherwise indicated. The NDV LaSota strain was obtained from ATCC and propagated in 9-day-old SPF chicken embryos. The velogenic strain of NDV, California 2002 (NDV/CA02; game chicken/US(CA)/S0212676/02), and the AMPV-C Colorado strain (AMPV-CO) were obtained from the pathogen repository bank at SEPRL, USDA-ARS (Athens, GA, USA). The virulent AMPV-C virus stock (AMPV-CO Tr) was prepared from the tracheal tissues of AMPV-CO strain-infected SPF turkeys and titrated in SPF turkeys to determine 50% infective dose (ID_50_) as described previously^16^. Viral RNA from the NDV-infected chicken embryo AF or DF-1 cells was extracted using the TRIzol-LS reagent according to the manufacturer’s instructions (Life Technologies). Total cellular RNA from tracheal tissues was extracted using a MagMAX™ AI/ND Viral RNA Isolation kit (Life Technologies) following the manufacturer’s procedures.

### Construction of recombinant LaSota cDNA clones containing the F and G genes of AMPV-C

The backbone of the previously generated infectious LaSota/AMPV-C G clone, pLS/AMPV-C G^13^, was used to construct a recombinant cDNA clone containing the F gene of AMPV-C. To facilitate the expression of the AMPV-C F gene, an internal ribosomal entry site (IRES) sequence was inserted between the open reading frames (ORF)s of the NDV F and AMPV-C F genes (Fig. 1) as described previously^27^. Briefly, the cDNA encoding the AMPV-C F ORF was subcloned downstream of the IRES sequence in pIRES-hrGFP-2a vector (pIRES, Clontech, Mountain View, CA). Subsequently, the IRES and the AMPV-C F ORF sequences were amplified and subcloned downstream of the NDV F ORF in the pLS/AMPV-C G vector using the In-Fusion PCR Cloning Kit (Clontech). The resulting recombinant clone contains the AMPV-C F gene as the 2^nd^ ORF in the NDV F gene transcription unit and the AMPV-C G gene as an independent transcription unit between the NDV F and HN gene. The full-length cDNA clone was designated pLS/AMPV-C F&G and amplified in Stbl2 cells at 30 °C for 24 hours and purified using a QIAprep Spin Miniprep kit (Qiagen).

### Virus rescue and propagation

Rescue of the recombinant, infectious LaSota/AMPV-C F&G virus was performed by transfecting the full-length cDNA clone, pLS/AMPV-C F&G, and supporting plasmids into HEp-2 cells as described previously^30^. The rescued virus was amplified by inoculating 100 µl of the infected cell lysate into the allantoic cavity of 9-day-old SPF chicken embryos and incubating the embryos at 37 °C. After 4 days of incubation, the AF was harvested and the presence of the rescued virus was detected by hemagglutination (HA) assay^31^. HA-positive AF was diluted in PBS and amplified in chicken embryos three times. The AF was harvested from the infected embryos, aliquoted, and stored at −80 °C as a stock. The nucleotide sequence of the rescued virus was determined by sequencing the RT-PCR products amplified from the viral genome as described previously^13^.

### Virus titration, pathogenicity, and growth dynamics assays

Titers of the LaSota, rLS/AMPV-C G, and rLS/AMPV-C F&G viruses were analyzed and compared by the standard HA test in a 96-well microplate, the 50% tissue culture infectious dose (TCID_50_) assay on DF-1 cells, and the 50% egg infective dose (EID_50_) assay in 9-day-old SPF chicken embryos^31^. Pathogenicity of the recombinant virus was also determined using the standard mean death time (MDT) and intracerebral pathogenicity index (ICPI) assays^31^.

### Immunofluorescence assay (IFA)

Expression of the AMPV-C F protein in infected DF-1 cells was examined by IFA using chicken anti-AMPV-C F polyclonal antibody. Briefly, confluent monolayers of DF-1 cells were infected with rLS/AMPV-C F&G at a multiplicity of infection (MOI) of 0.01. At 24 h post-infection, the infected cells were washed with phosphate buffered saline (PBS) and fixed with 10% zinc formalin (Fisher Scientific, Pittsburgh, PA) for 15 min at room temperature. The cells were permeabilized by adding 0.5% Triton X-100 (Sigma, St. Louis, MO) to the monolayer for 10 min at room temperature. The permeabilized cells were blocked with 5% goat serum (SouthernBiotech, Birmingham, AL) for 30 min at 37 °C. After blocking, the cells were stained with a mixture of chicken anti-AMPV-C F serum (a gift of Dr. Lizhong Luo from Canadian Food Inspection Agency, Canada) and mouse anti-NDV HN monoclonal antibody (Mab, a gift of Dr. Ron Iorio from University of Massachusetts Medical School, USA) for 1 hour at 37 °C. Cells were then washed with PBS and stained with a mixture of FITC-labeled goat anti-chicken IgG (H + L) (SouthernBiotech, 1:1000 dilution) and Alexa Fluor 568 conjugated goat anti-mouse IgG (Life Technologies, 1:1000 dilution) for 1 hour at 37 °C. Immunofluorescence of the infected cells was visualized and photographed using an inverted fluorescence microscope at 100X magnification with filter combinations for FITC or Alexa Fluor 568 (Nikon, Eclipse Ti, Melville, NY).

### Immunization and challenge experiment

To evaluate the efficacy of rLS/AMPV-C F&G vaccine against AMPV-C and NDV challenge, two animal experiments were performed. In experiment 1, SPF turkeys were used in the BLS-2E animal facilities at SEPRL to evaluate the protection against virulent AMPV-C challenge. In experiment 2, SPF turkeys were used in the BSL-3E animal facilities at SEPRL to evaluate the protection against lethal NDV challenge. All turkeys were housed in Horsfal isolators (Federal Designs, Inc., Comer, GA) with *ad libitum* access to feed and water. At the termination of the experiments, all birds were humanely euthanized in accordance with SEPRL’s Institutional Animal Care and Use Committee approved animal use protocols.

#### Experiment 1

Sixty one-day-old SPF turkey poults were randomly divided into six groups of 10 birds. Each bird in groups 1 and 2 was inoculated with 50 µl of the LaSota vaccine (10^7^ TCID_50_/ml) via intranasal (IN) and intraocular (IO) routes as vaccine vector controls for a total of 100 µl. Birds in groups 3 and 4 were vaccinated with 100 µl of rLS/AMPV-C G (1.0 × 10^7^ TCID_50_/ml) and birds in groups 5 and 6 were vaccinated with 100 µl of rLS/AMPV-C F&G (1.0 × 10^7^ TCID_50_/ml) per bird via IN/IO routes. At 14 days post-vaccination (DPV), the birds in groups 1, 3, and 5 were challenged with100 µl of pathogenic AMPV-C (1 × 10^3^ ID_50_/ml) via IN/IO routes. At 28 DPV, the birds in groups 2, 4, and 6 were challenged with the same dose of pathogenic AMPV-C via IN/IO routes. Immediately before challenge, blood samples were collected from each bird for detection of serum antibody responses. The challenged birds were monitored daily for clinical signs of AMPV disease for 14 days. Typical clinical signs of the AMPV disease, nasal exudates when squeezed (score 1), nasal discharge (score 2), and/or frothy eyes (score 3), were assessed using the scoring system of Cook *et al*.^17^. At 3, 5, and 7 days post-challenge (DPC), intra tracheal swabs were collected from each bird for detection of challenge virus shedding.

#### Experiment 2

Thirty one-day-old SPF turkeys were randomly divided into three groups of 10 birds. Birds were inoculated with 100 µl of PBS [Group 1], the LaSota vaccine (1.0 × 10^7^ TCID_50_/ml) [Group 2], rLS/AMPV-C G (1.0 × 10^7^ TCID_50_/ml) [Group 3], and rLS/AMPV-C F&G (1.0 × 10^7^ TCID_50_/ml) [Group 4] via IN/IO routes. At 14 DPV, the birds were challenged with a lethal dose of the NDV/CA02 virus as described previously^32^. Serum samples were collected immediately before challenge for NDV antibody detection using the standard hemagglutination inhibition (HI) assay^31^. After challenge, the birds were monitored daily for clinical signs of Newcastle disease and mortality for two weeks.

### Detection of immunoresponse and challenge virus shedding

The antibody response against NDV was determined by performing a standard HI assay using LaSota virus as antigen^17, 31^. The antibody response to AMPV-C was examined by an enzyme-linked immunosorbent assay (ELISA) as described previously^16^. Briefly, Sucrose gradient purified AMPV-C virus was used as antigen. Turkey sera were diluted (1:100) and individually tested in triplicates. Negative and AMPV-C positive sera were included in each plate of the ELISA test as controls. Turkey sera were deemed positive when the mean chemiluminescence relative light unit (RLU) value was greater than the mean RLU plus 2× standard deviation of the three dilutions (1:100–1:400) of the negative turkey serum pool. Viral shedding from turkey tracheal tissue following challenge with AMPV-C was detected by RT-PCR with AMPV-C N gene-specific primers as described previously^13, 16^.
